# Age-Related Changes in Femoral Head Trabecular Microarchitecture

**DOI:** 10.14336/AD.2018.0124

**Published:** 2018-12-04

**Authors:** Charlene Greenwood, John Clement, Anthony Dicken, Paul Evans, Iain Lyburn, Richard M. Martin, Nick Stone, Peter Zioupos, Keith Rogers

**Affiliations:** ^1^Cranfield Forensic Institute, Cranfield University, Shrivenham, UK.; ^2^Melbourne Dental School, University of Melbourne, Melbourne, Australia.; ^3^The Imaging Science Group, Nottingham Trent University, Nottingham, UK.; ^4^Cobalt Health, Cheltenham, UK.; ^5^Social and Community Medicine, Bristol University, Bristol, UK.; ^6^Physics and Astronomy, Exeter University, Exeter, UK.

**Keywords:** micro computed tomography (μ-CT), osteoporosis, aging, microarchitecture, trabecular bone, femoral head

## Abstract

Osteoporosis is a prevalent bone condition, characterised by low bone mineral density and increased fracture risk. Currently, the gold standard for identifying osteoporosis and increased fracture risk is through quantification of bone mineral density using dual energy X-ray absorption. However, many studies have shown that bone strength, and consequently the probability of fracture, is a combination of both bone mass and bone ‘quality’ (architecture and material chemistry). Although the microarchitecture of both non-fracture and osteoporotic bone has been previously investigated, many of the osteoporotic studies are constrained by factors such as limited sample number, use of ovariectomised animal models, and lack of male and female discrimination. This study reports significant differences in bone quality with respect to the microarchitecture between fractured and non-fractured human femur specimens. Micro-computed tomography was utilised to investigate the microarchitecture of femoral head trabecular bone from a relatively large cohort of non-fracture and fracture human donors. Various microarchitectural parameters have been determined for both groups, providing an understanding of the differences between fracture and non -fracture material. The microarchitecture of non-fracture and fracture bone tissue is shown to be significantly different for many parameters. Differences between sexes also exist, suggesting differences in remodelling between males and females in the fracture group. The results from this study will, in the future, be applied to develop a fracture model which encompasses bone density, architecture and material chemical properties for both female and male tissues.

Osteoporosis (OP) is a prevalent bone condition around the world, characterised by low bone mineral density and increased fracture risk. It is estimated that 1 in 3 women and 1 in 5 men are at risk of an osteoporotic fracture (https://www.iofbonehealth.org/facts-statistics). The probabilities increase proportionally with an aging population as osteoporosis is more likely to affect the elderly. The economic impact of osteoporosis is well known i.e. the UK’s National Health Service (NHS) spends > £2.0 billion per annum on OP hip fractures and their associated costs (https://thebms.org.uk/2010/10/breaking-point-osteoporosis-report/), but more impor-tantly it has a social impact resulting in the loss of mobility and an increased chance of mortality. Hip, vertebrae and wrist fractures are commonly associated with osteoporosis. In the year 2000, there were estimated 9 million new osteoporotic related fractures of which, 1.6 million were at the hip, 1.7 million were at the wrist and 1.4 were clinical vertebral fractures (www.iofbonehealth.org/facts-statistics). However, it is widely recognised that hip fractures lead to the largest risk of loss of independence and/or death [1]. Up to 20% of individuals die within the first year of a hip replacement, although pre-existing medical conditions often exist [2]. Of those individuals who survive, less than half regain their previous level of function [2]. As osteoporosis is symptomless, silent and progressive, the need for an accurate fracture prediction model is crucial for effective patient management. Currently, the gold standard for identifying osteoporosis and increased fracture risk is through quantification of bone mineral density (BMD) [3], using dual energy X-ray absorption (DEXA) [4]. However the use of BMD to diagnose osteoporosis is not without limitation, with a study by Wainwright reporting that 54% of new hip fractures occurred in women who did not have osteoporosis according to their BMD [5]. Thus, there is a need to identify other factors which influence bone strength and consequently fracture risk.

Literature suggests bone strength is a combination of bone density and ‘bone quality’[6-9], with bone quality referring to bone architecture (i.e. macro and micro) and material chemistry. Advances in technology, in particular micro computed tomography (μ-CT), have enabled new methods for accurately describing bone quality. For example, there have been a number of studies which investigate the change in trabecular microarchitecture with age using µ-CT [10-13]. These studies tend to have large specimen numbers (n > 90) but do not consider bone which has fractured due to diseases such as osteoporosis or osteoarthritis. A small number of studies (possibly due to the difficulty of obtaining human bone, especially osteoporotic specimens) have shown microarchitectural properties of bone potentially offer a superior way to differentiate between diseased bone which has fractured when compared to healthy controls (non - fractured tissue) [14-16]. Bone chemistry is more complex, with studies often providing contradicting results and conclusions [9, 17-21]. Unfortunately, many of the studies which investigate the quality of osteoporotic bone are limited by relatively low sample numbers, where n ≤ 15 for these studies [22-26]. Further, previous studies have often assumed a common remodelling mechanism for males and females. Thus groups of samples are not differentiated according to sex [10, 27, 28], which may lead to confounding findings. There is some recent evidence that, with aging, trabecular bone loss occurs through thinning of the trabeculae in males, whilst in females, perforation of trabeculae occurs, arguably this is due to sex hormone deficiencies [8, 29-32]. However, it may be argued this observation is due to the trabeculae initially being thinner in males and/or the resorption of the trabeculae occurs more aggressively and for longer in females due to menopause.

Using micro computed tomography (µ-CT), this study reports age-related microarchitectural changes in trabecular bone collected from the femoral head of 83 non-fracture individuals, within a wide age range of 20 - 93 years. The results differentiate between males and females, crucial for understanding any potential hormone dependent changes and developing a reliable fracture predictive model. Importantly, this study also compares non-fractured specimens to the microarchitecture of trabecular bone obtained from individuals who suffered a femoral neck fracture. The results provide an insight into quantified trabecular changes to bone modelling and remodelling caused not only by age and disease but also by sex. Previous CT studies have either examined mixed populations i.e. with no osteoporosis diagnosis [27, 33] or investigated osteoporotic tissue utilising specimens from females only [22-26, 34]. Other studies have exploited ovariectomised animal models [35-37] and often small osteoporotic sample numbers are employed [22, 25, 26, 38]. Therefore, we report herein the results of an aging study that enables a male/ female comparison, and also a fracture/non-fracture comparison for both males and females. Crucially, this is one of the first studies of its kind to compare male and female specimens from both non-fracture and fracture tissue, in order to investigate the potential modelling and remodelling differences between sexes. The findings of this work are fundamental to the development of new diagnostic tests for osteoporosis, as they provide a bench mark for the microarchitectural parameters which should be considered when developing a fracture risk model.

## MATERIALS AND METHODS

### Bone Specimens 

A sample set of 37 femoral heads were collected from osteoporotic patients who had suffered trauma fractures at the femoral neck and consequently required endoprosthetic hip replacement surgery. Ethical approval for the collection and use of these specimens was provided by Gloucestershire NHS trust REC (see acknowledge-ments). Non-fracture femoral head specimens were collected from 83 cadavers within the Melbourne Femur Collection. The donors died of natural or accidental causes, with no known diseases. Ethical approval for the collection and use of these specimens was provided by The University of Melbourne. The UK and Australian specimens were selected as they were the most accessible human samples in sufficient numbers to our research group. All individuals were Anglo-Celtic and followed western lifestyles. For both the fracture and non-fracture specimens, the donors were randomly selected. Population characteristics for both fracture and non-fracture specimens are provided in table 1.

**Table 1:** Population characteristics for fracture and non-fracture groups, differentiated according to sex.

	Fracture		Non - Fracture	
	Male	Female	Male	Female
Donors	7	30	44	39
Number of Specimens	23	58	44	39
Age Range (yrs)	74 - 84	59 - 91	21 - 93	20 - 90
Age Mean (yrs)	76.90 ± 2.72	82.47 ± 6.43	64.75 ± 19.00	66.18 ± 17.92
Weight Range (kg)	70 - 83	41 - 79	53 - 106	40 - 121
Weight Mean (kg)	76.36 ± 7.35	61.28 ± 8.95	78.59 ± 15.32	66.79 ± 19.77
Height Range (cm)	178 - 179	155 - 173	157 - 192	145 - 169
Height Mean (cm)	178.13 ± 0.65	163.91 ± 5.23	173.91 ± 8.53	159.63 ± 6.76

### Sample Preparation 

Trabecular bone was obtained and analysed for this study from the femoral head. Fracture and non-fracture specimens were obtained by a combination of trephine coring and mechanical cutting. The specimens were sampled randomly to avoid any orientational bias. Overall the strategy was to select random samples with respect to femoral head location although each sample was cut to include tissue from at least two quadrants of the head. For further information regarding sample preparation refer to [39]. The volume of the specimens ranged from approximately 1 - 3.5 cm^3^. Whilst archived the samples were stored at -70°C and were stored at -20°C during data collection. Prior to imaging, the specimens were cleaned using a high pressure warm water jet to remove bone marrow from within the trabecular spaces.

### Micro Computed Tomography (μCT)

The specimen microarchitecture was examined with micro computed tomography (μCT). Each specimen was scanned using a Nikon CT H225 (X-Tek Systems Ltd, Tring, Hertfordshire, UK) cone beam μCT scanner operated at 35 kV, and 115 μA. The geometric magnification produced a voxel dimension range of 15 - 25 μm for the fracture and non-fracture group specimens. Noise reduction and beam hardening corrections were applied to the data and VG Studio Max 2.2 (Volume Graphics GmbH, Heidelberg, Germany) utilised to visualise and quantify several microarchitectural features. These included trabecular thickness (TbTh), spacing (TbSp) and number (TbN), surface area (BS), material volume (BV) and total volume (TV). QRM µ-CT-HA (QRM GmbH, 91096 Möhrendorf, Germany) calibration phantoms, which differed in known volumetric tissue mineral density (_v_TMD) values, were scanned and reconstructed under the same conditions as the specimens. The mean grey scale values taken from the attenuation histograms for these phantoms were then used to construct a calibration curve of volumetric tissue mineral density (_v_TMD) values and grey scales. This allowed calculation of tissue mineral density values for the trabecular specimens. _v_TMD values were then used to determine volumetric bone mineral density values (_v_BMD) according to:-.

vBMD = vTMD ×BV/TV

_v_TMD refers to the density measurement restricted to within the volume of calcified bone tissue, and excludes any surrounding soft tissue, whereas _v_BMD is the combined density in a well-defined volume. BoneJ^©^ [40] was employed at a second stage to calculate additional microarchitectural parameters such as structure model index (SMI), which gives an indication of the trabecular geometry, with 0 suggesting a plate like structure, 3 signifying rod like trabeculae, 4 a sphere and < 0, concave. The authors recognise there are some limitations to SMI values as highlighted by Salmon et al. (2015). SMI calculations exploit the changes in surface curvature which occur as a structure varies from spherical, to cylindrical to planer [41]. SMI calculations are based on the assumption that the entire bone surface is convex and that the curvature differential is positive at all points on the surface. However, it has been argued that the intricate connections within the trabecular continuum could be predominantly concave in nature, which could potentially produce regions of negative differential [41]. This is highlighted by Salmon et al. who state ‘because SMI is set up to report a single figure summarising an entire bone surface, an SMI of 0 (ostensibly plate-like) can result from a surface that has an SMI of +1 on its convex portions and -1 on its concave portions’ [41].

### Statistical Analysis 

Linear regression analysis was carried out to statistically assess the correlation between the various microarchitecture parameters and age for the non-fracture group. The magnitude of the trend was estimated over a 5-year period. A general linear model ANOVA statistical analysis was also undertaken to determine significant differences between the parameters measured for age matched fracture and non-fracture groups. Anderson-Darling tests were carried out to determine whether the data values are normally distributed.

**Table 2:** Average values (in bold) and the associated errors (SEM) for the microarchitectural parameters for fracture and non-fracture groups.

	Fracture		Non - Fracture	
	Females	Males	Females	Males
BV/TV	0.18 ± 0.01	0.18 ± 0.01	0.30 ± 0.01	0.32 ± 0.01
BS/BV (mm^-1^)	16.06 ± 0.40	17.84 ± 0.53	10.83 ± 0.24	10.10 ± 0.22
TbTh (mm)	0.13 ± 0.004	0.11 ± 0.003	0.19 ± 0.004	0.20 ± 0.005
TbN (mm^-1^)	1.42 ± 0.03	1.60 ± 0.05	1.60 ± 0.02	1.57 ± 0.03
TbSp (mm)	0.60 ± 0.02	0.52 ± 0.02	0.44 ± 0.01	0.45 ± 0.01
SMI	1.81 ± 0.06	1.85 ± 0.07	1.14 ± 0.05	1.15 ± 0.06
BMD (g cm^-3^)	0.30 ± 0.01	0.31 ± 0.02	0.50 ± 0.02	0.52 ± 0.02
TMD (g HA cm^-3^)	1.61 ± 0.01	1.65 ± 0.01	1.64 ± 0.01	1.62 ± 0.01

The p-values calculated from the statistical analysis of the fracture and non-fracture parameters can be found in tables 3 and 4.

## RESULTS

**Figure 1.**
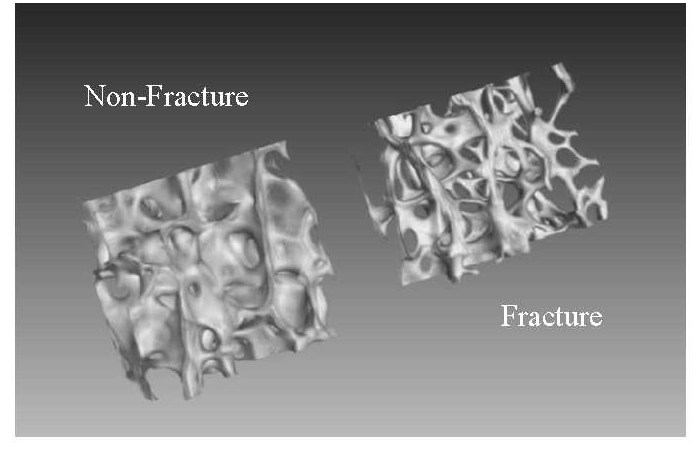
Micro-CT (µ-CT) three-dimensional rendered images from non-fracture (left) and fracture (right) female specimens of the same age (84 yrs).

Micro-CT (µ-CT) three-dimensional rendered images from each group, non-fractured and fractured, are provided in Figure 1. The average values and associated errors of the microarchitectural properties derived from the µ-CT reconstructions are presented in table 2. Results of the statistical testing applied to each characteristic parameter are summarised within tables 3 and 4. The p values and R^2^ values calculated from linear regression statistical analysis to quantify the correlation between the microarchitecture parameters and age for the non-fracture group, are provided in table 4. The rate of change in the parameters for the non-fracture group over a period of 5 years, are also provide in table 4.

### Volumetric Bone Mineral Density (_v_BMD)

**Figure 2.**
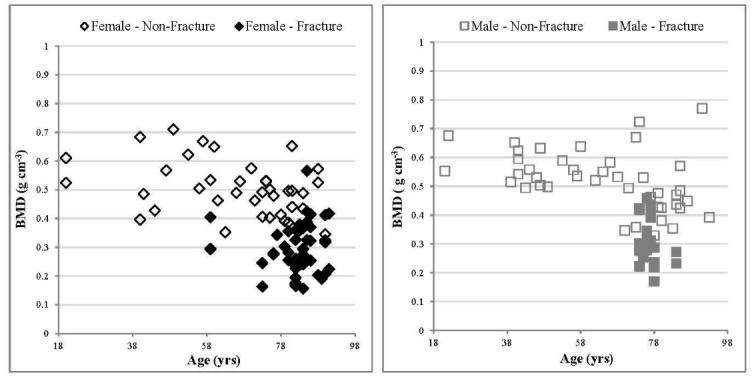
Relationship between BMD values and age, comparing fracture and non-fracture groups, female specimens (left) and male specimens (right). With age, a significant correlation was observed for both non-fracture males and females. The linear trend correlation coefficients for non-fracture males and females were p < 0.01, R^2^ = 0.19 and p < 0.05, R^2^ = 0.14 respectively. Errors have been excluded from the graphs for clarity. For the non-fracture group, each data point represents one donor. For the fracture group each data point represents an individual specimen several of which may arise from a single donor.

With age, the trend line correlation coefficients for BMD were relatively small (R^2 ^= 0.19 and R^2^ = 0.14 respectively for non-fracture males and females), but significant (p < 0.01) for both sexes (Fig. 2, Table 4). The gradient of the trend line indicated a decrease in BMD with age for non-fracture males and females, with a rate of change per 5 years of -0.012 ± 0.004 and -0.010 ± 0.004 g cm^-3 ^respectively (Table 4). No significant difference was observed between the two sexes in the non-fracture group. _v_BMD values for the fracture group were significantly lower (p < 0.01) than the non-fracture group for both sexes (Fig. 2, Table 3).

### Bone Volume Fraction (BV/TV)

As with BMD, the age trend line correlation coefficients for bone volume to total volume (BV/TV) were relatively small (R^2 ^= 0.19 and R^2^ = 0.22 respectively for non-fracture males and females), but significant (p < 0.01) for both sexes (Table 4). The gradient of the trend line indicates a decrease in BV/TV with age for non-fracture males and females, with a rate of change per 5 years of -0.007 ± 0.002 for both sexes (Table 4). No significant difference was observed between the two sexes in the non-fracture group. When age matched, BV/TV values for the fracture group were significantly lower (p < 0.01) than the non-fracture group for both sexes (Table 3). No significant difference between the fracture males and females was observed for BV/TV.

### Trabecular Thickness (TbTh)

**Figure 3.**
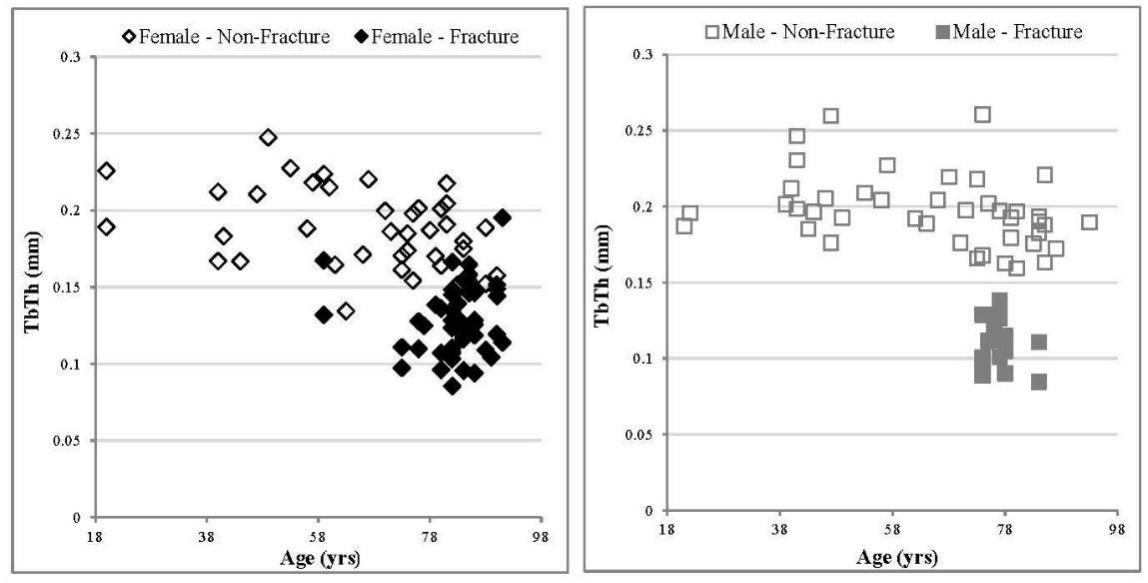
Relationship between TbTh values and age, comparing fracture and non-fracture groups, female specimens (left) and male specimens (right). There was no significant correlation with age for non-fracture males, whereas a significant correlation for non-fracture females was observed. The linear trend correlation coefficients for non-fracture females were p < 0.05, R^2^ = 0.13. Errors have been excluded from the graphs for clarity. For the non-fracture group, each data point represents one donor. For the fracture group each data point represents an individual specimen several of which may arise from a single donor.

No significant correlation between age and trabecular thickness (TbTh) was observed for the male specimens in the non-fracture group (p > 0.05). However, although the trend line correlation coefficients for TbTh were relatively small for non-fracture female specimens (R^2^ = 0.13), they were significant (p < 0.05) (Fig. 3, Table 4). The gradient of the trend line indicates a decrease in TbTh with age for non-fracture females, with a rate of change per 5 years of -0.003 ± 0.001 mm (Table 4). A significant difference was observed between the two sexes in the non-fracture group for TbTh (p < 0.05), with an average of 0.20 ± 0.005 mm for males and 0.19 ± 0.004 mm for females. The TbTh values for the fracture group are significantly lower for both sexes than the non-fracture group (p < 0.01) as reported in tables 2 and 3.

### Trabecular Number (TbN)

**Figure 4.**
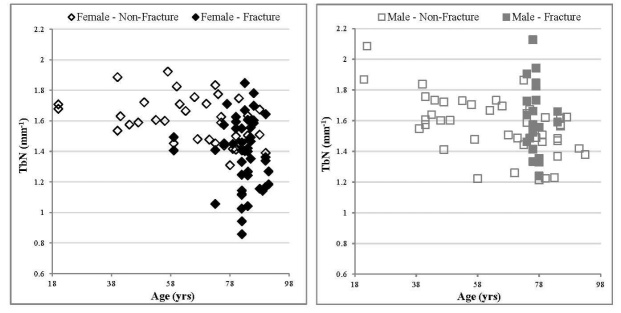
Relationship between TbN values and age, comparing fracture and non-fracture groups, female specimens (left) and male specimens (right). With age, a significant correlation was observed for both non-fracture males and females. The linear trend correlation coefficients for non-fracture males and females were p < 0.01, R^2^ = 0.19 and p < 0.05, R^2^ = 0.15 respectively. Errors have been excluded from the graphs for clarity. For the non-fracture group, each data point represents one donor. For the fracture group each data point represents an individual specimen several of which may arise from a single donor.

The relatively small correlation coefficients for non-fracture males and females (R^2 ^= 0.31 and R^2^ = 0.15 respectively) were significant (p < 0.05) for both sexes. (Fig. 4, Table 4). The gradient of the trend line indicates a decrease in TbN with age for non-fracture males and females, with a rate of change per 5 years of -0.028 ± 0.006 and -0.016 ± 0.006 mm^-1^ respectively (Table 4). No significant difference between the non-fracture males and females was observed. For this parameter, values for the female specimens in the fracture group were significantly lower than their equivalent age matched specimens in the non-fracture group (p < 0.05, Table 2 & 3). This was not the case for the males, where no significant difference was observed between the non-fracture and fracture groups.

**Table 3:** P-values for age matched ANOVA analysis of fracture (F) and non-fracture (NF) groups differentiated according to sex, for each microarchitectural parameter.

	ANOVA			
	Non - Fracture vs Fracture (Age Matched)			
	Male n = 21 (NF); n = 21 (F)		Female n = 22 (NF); n = 47 (F)	

	p - value	Mean difference	p - value	Mean difference
BV/TV	< 0.01	0.10 ± 0.02	< 0.01	0.10 ± 0.01
BS/BV (mm^-1^)	< 0.01	-7.15 ± 0.63	< 0.01	-4.81 ±0.51
TbTh (mm)	< 0.01	0.07 ± 0.01	< 0.01	0.05 ± 0.01
TbN (mm^-1^)	*	-0.12 ± 0.06	< 0.05	-0.15 ± 0.05
TbSp (mm)	*	0.03 ± 0.03	< 0.01	-0.14 ± 0.03
SMI	< 0.01	-0.60 ± 0.12	< 0.01	-0.60 ± 0.10
BMD (g cm^-3)^	<0.01	0.15 ± 0.03	< 0.01	0.18 ± 0.02
TMD (g HA cm^-3^)	*	-0.04 ± 0.02	< 0.05	0.05 ± 0.01

* indicates a p value > 0.05. n refers to the number of specimens. The mean difference between the non-fracture and fracture groups is also reported.

### Specific Surface Area (BS/BV) & Structure Model Index (SMI)

For the non-fracture male specimens, there was no significant correlation in specific surface area values, (bone surface / bone volume, BS/BV), with age (p > 0.05). Although the trend line correlation coefficients for BS/BV were relatively small for non-fracture female specimens (R^2^ = 0.11), they were significant (p < 0.05) (Table 4). The gradient of the trend line indicates an increase in BS/BV with age for non-fracture females, with a rate of change per 5 years of 0.136 ± 0.065 mm^-1^ (Table 4). Further, a significant difference in this parameter was observed between non-fracture males and females, with female specimens exhibiting on average, larger BS/BV values (p < 0.05, Table 2). BS/BV values for both males and females are significantly greater in the fracture group in comparison to the age matched non-fracture specimens (Table 2). This finding was also observed for the structure model index (SMI) values. No significant correlation was observed between SMI and age for the non-fracture groups (p > 0.05).

**Table 4:** P-values and R^2^ calculated from linear regression statistical analysis when comparing the various microarchitecture parameters and age for non-fracture males and females.

	Linear Regression Analysis					
	Non - Fracture Correlations with Age					
	Male			Female		

	p -value	R^2^	Δ (per 5 yrs)	p -value	R^2^	Δ (per 5 yrs)
BV/TV	< 0.01	0.19	-0.007 ± 0.002	< 0.01	0.22	-0.007 ± 0.002
BS/BV (mm^-1^)	*	0.04	*	< 0.05	0.11	0.136 ± 0.065
TbTh (mm)	*	0.01	*	< 0.05	0.13	-0.003 ± 0.001
TbN (mm^-1^)	< 0.01	0.31	-0.028 ± 0.006	< 0.05	0.15	-0.016 ± 0.006
TbSp (mm)	< 0.01	0.29	0.012 ± 0.002	< 0.01	0.23	0.009 ± 0.003
SMI	*	0.01	*	*	0.09	*
BMD (g cm^-3)^	< 0.01	0.19	-0.012 ± 0.004	< 0.05	0.14	-0.010 ± 0.004
TMD (g HA cm^-3^)	*	0.02	*	*	0.01	*

* denotes a p value > 0.05. For those parameters were a significant trend was observed, the rate of change (∆) per 5 years is also reported.

### Volumetric Tissue Mineral Density (_v_TMD) 

**Figure 5.**
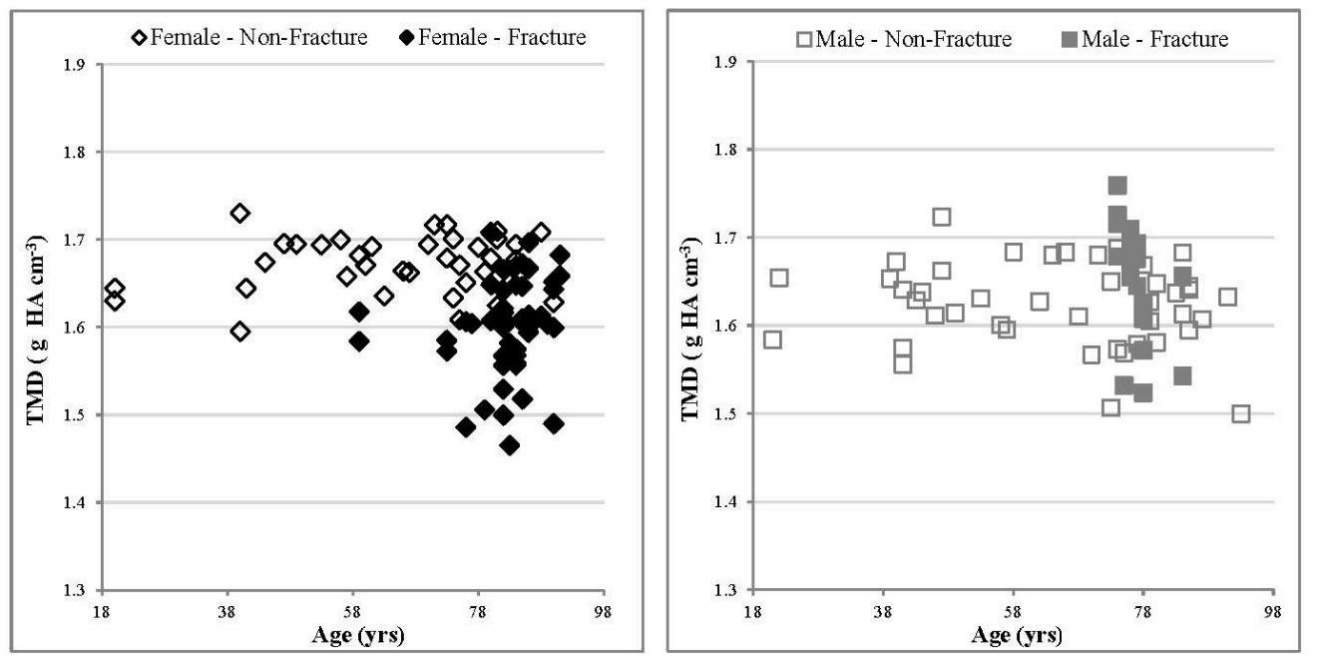
Relationship between TMD values and age, comparing fracture and non-fracture groups, female specimens (left) and male specimens (right). With age, no significant correlation was observed for non-fracture males and females (p > 0.05). Errors have been excluded from the graphs for clarity. For the non-fracture group, each data point represents one donor. For the fracture group each data point represents an individual specimen several of which may arise from a single donor.

For both males and females in the non-fracture group, there was no significant correlation in volumetric tissue mineral density (_v_TMD) values, (which provide density measurements restricted to the volume of calcified bone tissue), with age (Fig. 5, Table 4). No significant different was observed between the female and male non-fracture group (Tables 2 & 3). Interestingly, _v_TMD values from female specimens in the fracture group were found to be significantly lower than their age matched non-fracture counterparts (p < 0.05, Tables 2 & 3), whereas no significant difference were observed between the male groups.

The authors recognise an increased probability of type I errors in studies where large number of statistical tests are performed. However, this study mitigates against this by carrying out general linear model ANOVA analysis. As highlighted in table 3, all microarchitectural parameters are significantly different between age matched fracture and non-fracture females (p < 0.05); whilst for males all parameters except TbN, TbSp and _v_TMD are significantly different (p < 0.01).

### DISCUSSION

Overall, the characteristic values were similar to those presented previously for osteoporotic and non-fracture human tissues [10, 12, 22, 23, 25, 26, 33, 42]. A potential confounding element of the study was comparing tissue from UK donors to those of Australian donors. We carefully considered the legitimacy of comparing specimens from Australian and UK populations. All donors were Anglo-Celtic with a common western lifestyle. Unfortunately, no directly comparable previous UK non-fractured femoral head characteristics are available. However, we found no significant differences in mean values of CT characteristics between our Australian non-fracture group and a pooled population (3 US and 5 European) from previous studies of femoral head features [27, 33, 42-47]. Further, the incidence of hip fracture is the same in both UK and Australian populations. Although age alone was considered for this study, there could potentially be other uncontrolled confounding influences such as body mass index (BMI), calcium supplements, diet and physical activity. Future work investigating the affect these confounders may have on the microarchitecture is required.

The values for _v_BMD presented in this study agree with previous studies [48, 49]. Repeated findings are that _v_BMD decreases with age due to loss of trabeculae and osteoporotic patients have significantly lower _v_BMD values than their non-fracture counterparts, due to the loss of mineral mass [50]. Although BMD measured by DEXA is currently the gold standard for determining fracture risk, the magnitude of _v_BMD population variability at any age (see Fig. 2) strongly indicates why 54% of new hip fractures occur in woman with normal BMD values [5]. For example, several individuals within the non-fracture group have a significantly lower _v_BMD than the mean for that age group. This highlights the need for a more comprehensive fracture prediction model which incorporates microarchitecture parameters associated with bone.

This study supports the generally held view that bone loss is observed with increasing age [10, 12, 48]. The architectural parameters reported here suggest that this is caused by a steady loss and/thinning of the trabeculae within non-fracture patients. With increasing age, trabeculae become more rod like in geometry as indicated by increasing SMI values, assuming that this material system acts conventionally as predicated by SMI (see previous section regarding the limitations of SMI analysis), resulting in a greater specific surface area (>BS/BV). The majority of the microarchitecture properties between non-fracture males and females were not significantly different. Significant differences were observed between TbTh and specific surface area (BS/BV), with the males having slightly greater TbTh values and consequently lower BS/BV values than the females. This may suggest remodelling of bone differs between sexes for the non-fracture specimens when considering TbTh and BS/BV only and/or differences are too small to be detected given the level of precision employed. This is in contrast to a view proposed by recent studies where trabecular bone loss with age is thought to occur predominately through thinning of the trabeculae and reduced bone formation in males, whilst in females, perforation and complete removal of the trabeculae occurs leading to a loss of connectivity between trabeculae [8, 30-32]. However, this trend was observed in the fracture group specimens. This is consistent with previous results where a decline in TbN was observed in woman at the onset of menopause, which consequently caused an overall decline in estimated bone strength [13].

The fracture group microarchitecture parameters were in most cases significantly different to those of the age matched non-fracture group. The results, as previously reported [14, 15], show the loss of bone is significantly greater in the fracture groups. As reported elsewhere [51], the loss of bone appears to occur predominately through thinning of the trabeculae for the fracture male specimens, without a significant loss in the number of trabeculae. In contrast, for the fracture female specimens thinning of the trabeculae results in a reduction in the number of trabeculae. This suggests the remodelling of osteoporotic bone may differ according to sex within the fracture group. It is proposed that the origin of such a difference could be influenced by or be caused by fundamental differences in bone mineral chemistry. Further, the results suggest that in order to provide an accurate fracture predication model based on _v_BMD and microarchitecture parameters, it may be crucial to develop two models; one for females and one for males.

The volumetric tissue mineral density (_v_TMD) values, calculated from the grey scale attenuation of the µ-CT images, may be consistent with a change in bone chemistry. Many studies have investigated various parameters associated with bone mineral chemistry, although many report conflicting results. For example, hydroxyapatite (HA) crystallite size has been reported to increase [17, 24, 52, 53], decrease [39, 54] or remain constant [20] with age and in osteoporotic tissue. For the non-fracture group, this study did not demonstrate any change of _v_TMD with age. This may suggest that either there is no change in bone chemistry with age, or the changes are below the µ-CT detection limit for _v_TMD. Crucially, _v_TMD values for the female fracture group are significantly lower than that of the non-fracture female group. It is proposed that this is due to a change in the mineral physico-chemical properties, rather than a reduction of the mineral/collagen ratio. For example, a change in the amount of carbonate, which accounts for 4 - 8 wt. % of bone mineral [55], would modify _v_TMD measured values. In particular, an increase in B - type carbonate substitution (carbonate for phosphate), would result in a lower tissue mineral density. The density of stoichiometric HA has been reported as ~ 3.2 g cm^-3 ^[56], and literature has shown this to decrease with the incorporation of increasing amounts of carbonate [57, 58]. Other ions can also substitute into the apatite lattice, causing changes to density, for example fluoride (F^-^), but these substitutions are thought to be at a much lower level than carbonate. This is consistent with previous research which reported a significant increase in the carbonate content of fracture tissue when compared to age matched non-fracture specimens [39].

Conversely, no significant difference was observed between the fracture male specimens and the non-fracture specimens when considering the _v_TMD values. However, the _v_TMD is significantly greater for the fracture male specimens than the female fracture specimens, which may be due to a change in the mineral chemistry and/ or the organic component in bone. A significantly smaller amount of collagen has previously been observed in osteoporotic specimens [18, 59, 60], which may result in an increase in tissue density. The density of collagen is lower than that of bone mineral, therefore a change in mineral to organic content could result in an average increase in _v_TMD values. Further analysis would need to be carried out to investigate the organic content, with the hypothesis that the male fracture specimens would exhibit a significant difference in the mineral to organic content compared to fracture female specimens.

The differences in the _v_TMD values between males and females in the fracture group, as previously hypothesised, maybe due to remodelling differences caused by different hormonal changes in men and women [8, 29-32]. These remodelling differences may indicate two distinct mechanisms for the formation of OP tissue. Male OP sufferers potentially exhibit a change in the mineral to organic content, leading to loss in ductility and an increase in mineral brittleness whereas, for female OP sufferers, a physico-chemical change in the mineral occurs due to an increase in carbonate content.

### Conclusion

The main aim of this study was to provide an insight into quantified trabecular changes caused not only by age and disease but also by sex. The microarchitectural properties of trabecular bone obtained from fracture and non-fracture groups, which were differentiated according to sex, were investigated for this study. As shown through statistical analysis, all of the microarchitectural parameters for females (when age matched) were significantly different between fracture and non-fracture groups*.* This was also the case for the male specimens, with the exception of TbN, TbSp and _v_TMD, were no significant differences were observed. This may suggest for the fracture group, differences in remodelling between males and females. It has been proposed that this is due to material quality/ chemistry differences between the two sexes, collaborated to a certain extent by _v_TMD values. This study has also shown with age for both sexes, there is a progressive loss of bone mineral, through thinning and loss of trabeculae, although the extent of loss is significantly less than the fracture group.

This study is consistent with previous studies and suggests analysis of bone microarchitecture may provide a more robust non - invasive fracture prediction for osteoporosis diagnosis. However, currently the resolution of high resolution CT (HR-CT) used for *in vivo* analysis of living patients is lower than that of µ-CT and further work would be required to translate this *ex vivo* study through to *in vivo* examination using the critical parameters. Crucially, this study suggests that changes in fracture tissue are not just associated with progressive age-related deterioration in bone, with significant differences existing in the microarchitecture between age-matched non-fracture and fracture groups. This is possibly due to remodelling differences, which could potentially be investigated through bone chemistry/ quality parameters.
